# Efficacy and toxicity of image-guided intensity-modulated radiation therapy combined with dose-escalated brachytherapy for stage IIB cervical cancer

**DOI:** 10.18632/oncotarget.22434

**Published:** 2017-11-15

**Authors:** Weiping Wang, Qingyu Meng, Xiaorong Hou, Xin Lian, Junfang Yan, Shuai Sun, Zhikai Liu, Zheng Miao, Dunhuang Wang, Xiaoliang Liu, Ke Hu, Fuquan Zhang

**Affiliations:** ^1^ Department of Radiation Oncology, Peking Union Medical College Hospital, Chinese Academy of Medical Sciences and Peking Union Medical College, Beijing, P.R. China

**Keywords:** cervical cancer, image-guided radiation therapy, intensity-modulated radiation therapy, dose-escalated brachytherapy, FIGO stage IIB

## Abstract

Considering internal organ motion and tumor regression, it is controversial to use intensity-modulated radiation therapy (IMRT) in definitive radiotherapy for cervical cancer. In this study, we evaluated the efficacy and toxicity of IMRT combined with dose-escalated intracavitary brachytherapy (ICBT) for cervical cancer. In total, 373 consecutive FIGO-stage-IIB cervical cancer patients treated with IMRT combined with ICBT and concurrent chemotherapy were included in this study. A dose of 50.4 Gy in 28 fractions was delivered to the pelvis for IMRT. Weekly cone-beam computed tomography or daily megavoltage computed tomography was used for image guiding. For ICBT, 30–36 Gy in five to seven fractions were prescribed to point A. All patients received concurrent chemotherapy. The median follow-up duration was 32.5 months (range, 3.1–119.8 months). The three-year overall survival, disease-free survival and local control rates were 87.5%, 82.2% and 92.5%, respectively. Sixty patients (16.1%) experienced treatment failure, including 23 patients (6.2%) with pelvic relapse. The incidences of ≥grade 3 chronic gastrointestinal and genitourinary toxicity were 2.7% and 2.4%, respectively. These findings indicate that image-guided IMRT combined with dose-escalated ICBT results in good survival with acceptable toxicity in stage IIB cervical cancer patients.

## INTRODUCTION

Cervical cancer is the seventh-most-common cancer for women in China, and it was estimated that there were 98.9 thousand new cases and 30.5 thousand deaths in 2015 [1]. At present, the standard treatment approach for locally advanced cervical cancer is concurrent chemoradiotherapy (CCRT). Traditionally, external beam radiation therapy (EBRT) has been delivered via conventional radiotherapy or conformal radiation therapy (CRT), with anteroposterior and posteroanterior parallel portals or four-field “box” radiotherapy. These approaches deliver large irradiation volumes and cause considerable toxicity to the bladder, rectum and bowel. On the other hand, intensity-modulated radiation therapy (IMRT) can reduce the dose to organs at risks (OARs) while achieving a comparable or better dose distribution to the clinical target volume (CTV). The Radiation Therapy Oncology Group 0418 trial demonstrated that IMRT reduced the toxicity of radiation therapy without worsening disease control for postoperative International Federation of Gynecology and Obstetrics (FIGO) stage IA-IIB cervical cancer patients [2, 3]. IMRT is currently widely used for postoperative cervical cancer patients.

Considering internal organ motion and tumor regression [4–8], it is controversial to use IMRT in definitive radiotherapy for cervical cancer. Image-guided radiation therapy (IGRT) allows adjustment and correction of the radiation beam or the patient’s position, and thus is a more accurate form of dose delivery for patients. IGRT may reduce the probability of a geographic miss during treatment delivery for cervical cancer patients.

As it is for other locally advanced cervical cancers, CCRT is the standard treatment approach for stage IIB cervical cancer. However, uterine and cervical motion may be greater in stage IIB cervical cancer patients than in stage IIIB patients, as the uteri and cervixes of the latter are usually fixed due to wide parametrial involvement. Thus, it is riskier to use IMRT in stage IIB cervical cancer.

For stage IIB cervical cancer, previous studies have demonstrated that doses to point A greater than 85 Gy were associated with better central control [9, 10]. Further increase in the dose to point A beyond 85Gy was not associated with improved central control, but rather correlated with additional complications [9]. After definitive radiotherapy, the local recurrence rate of stage IIB cervical cancer has ranged from 12.41–19.9% [11–13], which is still too high.

At our institute, we began to treat cervical cancer patients with image-guided IMRT in 2005. The dose to OARs is lower with IMRT than with CRT or conventional radiotherapy, allowing us to deliver higher doses to point A with intracavitary brachytherapy (ICBT, 30–36 Gy in five to seven fractions) for better local control with acceptable toxicity. Further, high-dose ICBT can compensate for the insufficient dose to the tumor caused by the potential geographic miss of the target volume during IMRT. In this study, we retrospectively analyzed the survival and toxicity of FIGO stage IIB cervical cancer patients treated with image-guided IMRT combined with high-dose ICBT and concurrent chemotherapy.

## RESULTS

### Patients’ characteristics and treatment

In total, 373 patients were eligible for this study. The median age of the patients was 50 years (range, 24–73 years). The majority of patients (336/373) had squamous cell carcinoma. Ninety-two patients (24.7%) had regional lymph node metastases (LNM), including 74 patients with pelvic LNM, one patient with para-aortic LNM, and 17 patients with pelvic and para-aortic LNM. Fixed-field IMRT (FF-IMRT), volumetric modulated arc therapy (VMAT) and helical tomotherapy (HT) were used in 124 (33.2%), 232 (62.2%) and 17 (4.6%) patients, respectively. Fifty-two patients (13.9%) received extended-field radiotherapy. Dosages ≥30 Gy to point A by ICBT were prescribed for 343 (92.0%) patients. One patient did not receive ICBT because of cervical atresia, so a boost dose of 20 Gy in 10 fractions was delivered to the cervix with VMAT after whole pelvic irradiation. The concurrent chemotherapy regimen was cisplatin for 346 patients (92.8%), and 318 patients (85.3%) received four cycles or more of concurrent chemotherapy. Four patients did not complete the radiotherapy. One of them died of acute renal failure after 5.4 Gy in three fractions of pelvic EBRT and one cycle of cisplatin chemotherapy. The other three patients did not complete radiotherapy because they refused to continue ICBT (two patients received 6 Gy in one fraction and one patient received 12 Gy in two fractions). The detailed characteristics are shown in Table 1.

**Table 1:** Patients, tumor and treatment characteristics

	Characteristic	No. of patients	Percentage (%)
Age (years old)	Median	50	
	<65	353	94.6
	≥65	20	5.4
Histology	Squamous cell carcinoma	336	90.1
	Adenocarcinoma	28	7.5
	Adenosquamous carcinoma	6	1.6
	Undifferentiated carcinoma	2	0.5
	Neuroendocrine carcinoma	1	0.3
Lymph nodes metastasis	Regional lymph nodes metastasis	92	24.7
	Pelvic lymph nodes metastasis	91	24.4
	Para-aortic lymph nodes metastasis	18	4.8
EBRT technique	FF-IMRT	124	33.2
	VMAT	232	62.2
	HT	17	4.6
Extended field irradiation	Yes	52	13.9
	No	321	86.1
Concurrent chemotherapy	Cisplatin	346	92.8
	Paclitaxel	27	7.2
Concurrent chemotherapy	≥4 cycles	318	85.3
	<4 cycles	55	14.7
Dose of intracavitary brachytherapy	≤30 Gy	30	8.0
	30–36 Gy	291	78.0
	>36 Gy	52	13.9

EBRT indicates external beam radiation therapy; FF-IMRT, fixed field intensity modulated radiation therapy; VMAT, volumetric modulated arc therapy; HT, helical tomotherapy.

### Outcomes and patterns of failure

**Figure 1:**
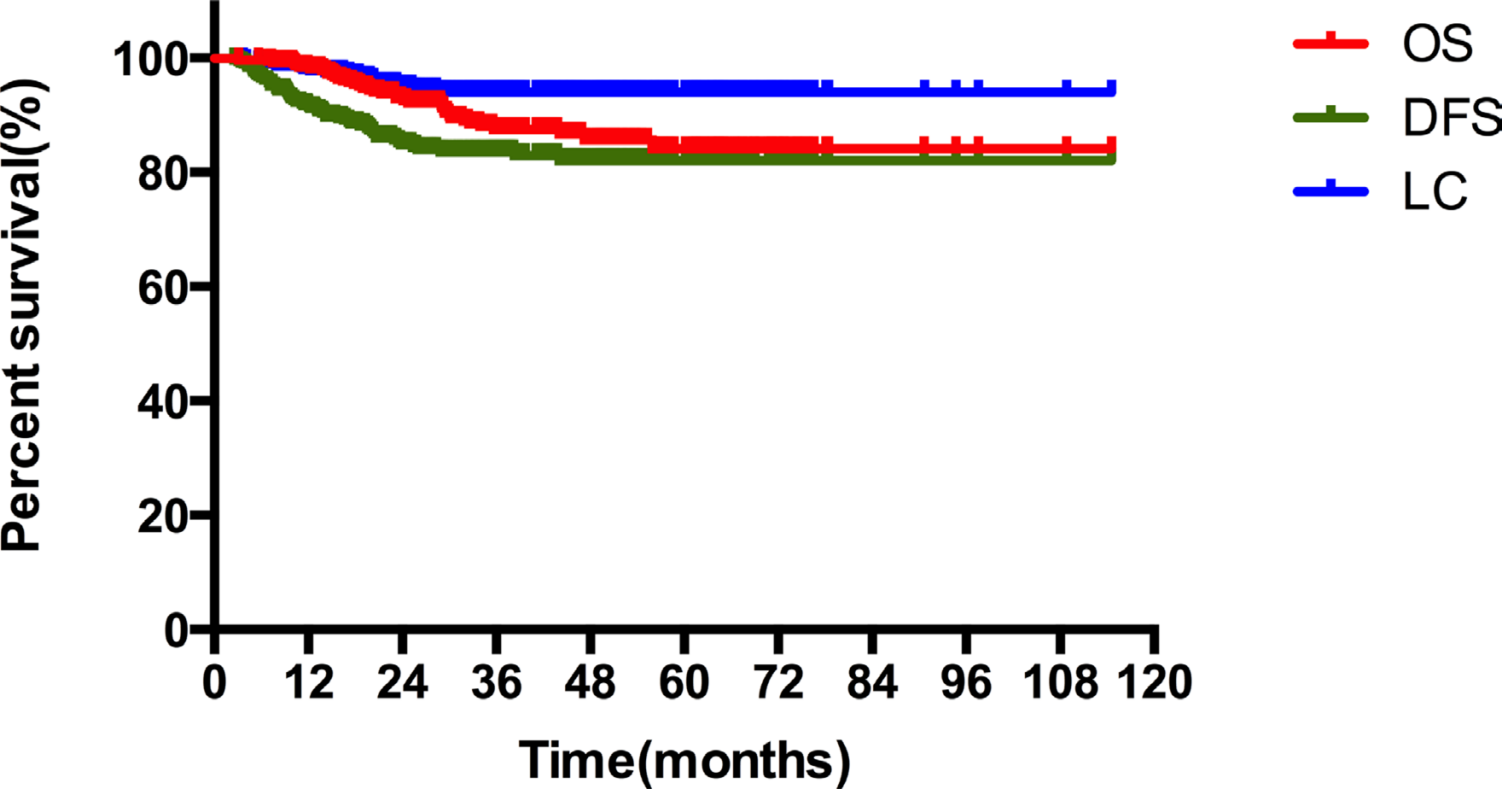
The overall survival (OS), disease-free survival (DFS) and local control (LC) rates of stage IIB cervical cancer patients

The median follow-up duration was 32.5 months (3.1–119.8 months). The three-year overall survival (OS), disease-free survival (DFS) and local control (LC) rates were 87.5%, 82.2% and 92.5%, respectively. The estimated five-year OS, DFS and LC rates were 84.1%, 80.7% and 92.5%, respectively (Figure 1).

Sixty patients (16.1%) experienced treatment failure, including 21 patients (5.6%) with pelvic relapse, 37 patients (9.9%) with distant metastasis, and two patients (0.5%) with both pelvic relapse and distant metastasis. The most common sites of pelvic relapse were the cervix or parametrium (12 patients, 3.2%) and the vagina (seven patients, 1.9%). Twenty-five patients (6.7%) experienced metastasis of the lung, which is the most common site of distant metastasis. Other common sites of distant metastasis included the para-aortic lymph nodes (nine patients, 2.4%) and the mediastinal or cervical lymph nodes (six patients, 1.6%).

Of the 92 patients with regional LNM, seven patients (7.6%) experienced regional lymph node failure. Of the 74 patients with positive pelvic lymph nodes and negative para-aortic lymph nodes, four patients (5.4%) experienced lymph node failure, including one with residual disease and three with recurrence. Three of the 18 (16.7%) patients with para-aortic LNM had regional lymph node failure, including one patient with residual lymph node disease and two patients with lymph node recurrence.

Thirty-eight patients (10.2%) had died by the end of follow-up; the median survival of the deceased patients was 20.0 months (range, 3.1–55.9 months). Thirty-five of them died of cervical cancer and one died of acute treatment toxicity. Two patients died for other reasons: one patient died of peritoneal mesothelioma and one patient died of heart failure.

### Prognostic factors

Age, histology, tumor size, pelvic LNM, para-aortic LNM, squamous cell carcinoma antigen (SCC Ag) levels, hemoglobin levels and radiotherapy duration were evaluated in univariate analysis. As shown in Table 2, the tumor size and hemoglobin level were significant prognostic factors for OS, DFS and LC. An age ≥65 years, pelvic LNM and para-aortic LNM were associated with worse OS and DFS. Patients with SCC Ag levels ≥10 ng/mL had worse DFS and LC.

**Table 2:** Unvariate analysis of factors influencing OS, DFS and LC

Factors	No. of patients	3-year OS (%)	*p*	3-year DFS (%)	*p*	3-year LC (%)	*p*
Age (years old)							
<65	353	88.9	0.0015	83.2	0.0351	92.9	0.3986
≥65	20	53.5		57.8		81.8	
Histology							
Squamous cell carcinoma	336	87.7	0.4933	83.1	0.2738	92.6	0.9209
Non-Squamous cell carcinoma	37	87.2		72.4		90.4	
Tumor size							
<5 cm	238	91.3	0.0030	87.9	0.0005	96.6	0.0002
≥5 cm	135	80.8		72.2		85.2	
Pelvic lymph nodes metastasis							
No	282	89.2	0.0348	86.1	0.0012	93.8	0.1098
Yes	91	82.4		70.3		88.5	
Para-aortic lymph nodes metastasis							
Yes	355	89.1	<0.0001	84.8	<0.0001	92.8	0.2896
No	18	56.4		32.4		88.5	
SCC Ag							
<10 ng/ml	198	92.0	0.0825	90.4	<0.0001	96.6	0.0006
≥10 ng/ml	87	84.6		68.4		83.6	
Hemoglobin levels							
≥110 g/L	301	90.6	0.0020	85.7	0.0002	94.6	0.0033
<110 g/L	72	76.3		67.8		84.0	
Radiotherapy duration							
≤8 weeks	295	88.0	0.5853	83.1	0.2784	92.7	0.6016
>8 weeks	78	85.6		78.5		91.8	

OS indicates overall survival; DFS, disease-free survival; LC, local control; SCC Ag, squamous cell carcinoma antigen.

The results of multivariate analysis are shown in Table 3. Older ages were associated with worse OS (*p <* 0.001, 95% confidence interval [CI]: 2.687–25.405) and DFS (*p =* 0.047, 95% CI: 1.014–8.160). Patients with bulky tumors (≥5 cm) had worse OS (*p =* 0.019, 95% CI: 1.151–4.749), DFS (*p =* 0.002, 95% CI: 1.366–4.182) and LC (*p =* 0.003, 95% CI: 1.654–11.598) than those with tumors <5 cm. Para-aortic LNM was associated with worse OS (*p =* 0.011, 95% CI: 1.414–14.414) and DFS (*p =* 0.001, 95% CI: 1.749–10.203). Patients with SCC Ag levels ≥10 ng/mL had worse OS (*p =* 0.007, 95% CI: 1.332–6.358), DFS (*p =* 0.001, 95% CI: 1.563–5.354) and LC (*p =* 0.032, 95% CI: 1.093–7.641) than those with SCC Ag levels <10 ng/mL. A low hemoglobin level was an independent prognostic factor for OS (*p =* 0.027, 95% CI: 1.097–4.642).

**Table 3:** Multivariate analysis of factors influencing OS, DFS and LC

Factors	OS		DFS		LC	
	HR (95% CI)	*p*	HR (95% CI)	*p*	HR (95% CI)	*p*
Age (<65 vs. ≥65)	8.261 (2.687–25.405)	<0.001	2.877 (1.014–8.160)	0.047	3.728 (0.837–16.569)	0.084
Tumor size (<5 cm vs. ≥5 cm)	2.338 (1.151–4.749)	0.019	2.390 (1.366–4.182)	0.002	4.379 (1.654–11.598)	0.003
Pelvic lymph nodes metastasis (No vs. Yes)	1.310 (0.557–3.079)	0.536	1.358 (0.717–2.572)	0.347	1.644 (0.628–4.303)	0.311
Para-aortic lymph nodes metastasis (No vs. Yes)	4.515 (1.414–14.414)	0.011	4.224 (1.749–10.203)	0.001	1.688 (0.338–8.438)	0.524
SCC Ag (<10 ng/ml vs ≥10 ng/ml)	2.910 (1.332–6.358)	0.007	2.892 (1.563–5.354)	0.001	2.890 (1.093–7.641)	0.032
Hemoglobin (≥110 g/L vs <110 g/L)	2.257 (1.097–4.642)	0.027	1.660 (0.924–2.983)	0.090	1.538 (0.627–3.774)	0.348

OS indicates overall survival; DFS, disease-free survival; LC, local control; SCC Ag, squamous cell carcinoma antigen; HR, hazard ratio; CI, confidence interval.

### Toxicity

Sixty-one patients (16.4%) developed ≥grade 2 chronic gastrointestinal toxicity, and 10 of them (2.7%) experienced ≥grade 3 gastrointestinal toxicity (Table 4). Forty-two patients (11.3%) developed ≥grade 2 chronic genitourinary toxicity, and eight of them (2.1%) experienced ≥grade 3 genitourinary toxicity. A total of 12 patients (3.2%) experienced ≥grade 3 chronic toxicity, including two patients with grade 3 enteritis, three patients with grade 3 cystitis, three patients with rectovaginal fistula, three patients with bowel obstruction, two patients with uterorectal fistula, one patient with sigmoid fistula, and one patient with ureteral obstruction.

**Table 4:** Chronic toxicity of stage IIB cervical cancer patients treated with IMRT

	Grade 2	Grade 3	Grade 4	Grade 5
Gastrointestinal toxicity	51 (13.7%)	5 (1.3%)	5 (1.3%)	0 (0)
Genitourinary toxicity	34 (9.1%)	5 (1.3%)	3 (0.8%)	0 (0)

IMRT indicates intensity modulated radiation therapy.

## DISCUSSION

IMRT allows highly conformal dose delivery to the target volume and a high dose gradient out of the target volume. Therefore, IMRT delivers a significantly lower dose to the bladder, rectum, bowel and bone marrow than four-field conformal pelvic radiotherapy for cervical cancer patients, without compromising target coverage [14, 15]. However, the uterus, cervix and OARs are prone to positional changes over time. The positions of organs during treatment may differ from their positions at the time of simulation and planning. Treating cervical cancer patients with definitive IMRT is risky, and sufficient margins should be added to the target volume. Van de Bunt *et al.* performed weekly magnetic resonance imaging (MRI) for 20 cervical cancer patients, and demonstrated that the CTV (including the gross tumor volume [GTV], the entire uterus, at least the upper 1/3 of the vagina and the parametrial tissue) required margins of 24, 17, 12, 16, 11 and 8 mm in the anterior, posterior, right lateral, left lateral, superior and inferior directions, respectively [4]. Chan *et al.* found that margins of 4 cm at the uterine fundus and 1.5 cm at the cervical os were required to encompass 90% of the interscan motion [5]. Large CTV margins may increase the dose to OARs and decrease the disadvantages of IMRT. IGRT is one way to safely reduce the margins. At our institute, an 8–10 mm margin was added to the CTV and an additional 5–10 mm margin was added to the vagina, cervix and uterus. Weekly cone-beam computed tomography (CBCT) or daily megavoltage CT (MVCT) was used to determine necessary position corrections. The local failure rate (6.2%) was low and the toxicity was acceptable. This indicates that these margins combined with our IGRT approach are feasible for cervical cancer patients treated with IMRT.

IMRT can cause less treatment toxicity than anteroposterior and posteroanterior parallel portals or four-field “box” radiotherapy, while achieving comparable or better treatment efficacy. Kidd *et al.* treated 452 cervical cancer patients with curative-intent radiotherapy (135 with positron emission tomography (PET)/CT-guided definitive IMRT and 317 with non-IMRT). The OS and cause-specific survival were better in the IMRT group (*p <* 0.0001). Cox multivariate analysis revealed that the treatment technique (IMRT vs. non-IMRT) was an independent factor predicting cause-specific survival (*p =* 0.0002). The incidences of ≥grade 3 bowel or bladder toxicity were 6% and 17% for patients treated with IMRT and non-IMRT, respectively (*p =* 0.0017) [16]. Du *et al.* reported that the estimated five-year progression-free survival rate was significantly higher for IMRT patients than for conventional radiotherapy patients (64.9% vs. 44.3%, *p =* 0.031). The IMRT patients also experienced significantly lower acute and chronic toxicities than the conventional radiotherapy patients [17]. A prospective randomized study from India included 44 patients with stage IIB-IIIB SCC of the cervix (22 IMRT and 22 CRT). The IMRT and CRT groups had similar OS and DFS, while patients in the IMRT group were less likely to experience ≥grade 3 acute gastrointestinal toxicity (4.5% vs. 27.3%, *p =* 0.047) and chronic gastrointestinal toxicity (13.6% vs. 50%, *p =* 0.011) [18].

The three-year to five-year OS rates have been reported to be 63.1–86.8% for FIGO stage IIB cervical cancer patients [9, 11–13, 19, 20]. In our study, the estimated five-year OS rate was 84.1%. The incidences of ≥grade 3 gastrointestinal and genitourinary toxicity for cervical cancer patients treated with non-IMRT have been reported to be 4.14–18.4% and 0–15%, respectively [11, 12, 17, 19]. In our study, the incidences of grade 3 or higher gastrointestinal and genitourinary toxicity were 2.7% and 2.4%, respectively. These incidences were lower than most of those previously reported for non-IMRT. It is worth noting that for 92% of our patients, the dose of ICBT was 30 Gy or greater. The estimated cumulative dose to point A in these patients was greater than 90 Gy (equivalent dose in 2-Gy fractions [EQD2], α/β = 10), which was higher than those in previous studies [11, 12, 19, 21]. Thus, with IMRT, we delivered a higher dose to point A, with a comparatively low incidence of ≥grade 3 toxicity.

In the last two decades, MRI- and CT-based image-guided brachytherapy (IGBT) have been of increasing interest. IGBT was reported to improve the survival of cervical cancer patients, while causing lower morbidity than 2D brachytherapy [22–24]. In the RetroEMBRACE study, 731 cervical cancer patients from 12 institutions were treated with IGBT, and the median follow-up duration was 43 months. The mean D90 (dose delivered to 90% of the target volume) of the high-risk clinical target volume was 87 ± 15 Gy (EQD2). The grade 3–5 bladder and gastrointestinal morbidity were 5% and 7%, respectively. The three-year LC and OS rates were 91% and 74% overall, respectively, and were 93% and 78% for stage IIB patients [22]. Thus, the three-year LC rate was similar to that in our study (92.5%). Patients in the RetroEMBRACE study achieved comparable LC to the patients in our study, but with a lower radiotherapy dose and less concurrent chemotherapy (77.4%) [22]. However, due to the heavy cervical cancer burden [1] and economic factors, IGBT is not widely used in developing countries at present. Thus, 2D brachytherapy may be the main treatment approach for cervical cancer for a long period in developing countries like China. At our institute, we make a 2D brachytherapy plan after every insertion and conduct a CT scan after the first insertion. We do our best to deliver a sufficient dose to the tumor and to reduce the toxicity. Considering the high LC and acceptable toxicity associated with our brachytherapy approach, this approach may be suitable for developing countries.

Van de Bunt *et al.* reported that the primary GTV of cervical cancer patients decreased by 46% (range, 6.1–100%) on average after about 30 Gy EBRT. Second IMRT plans spared the rectum at the 95% dose level of the prescribed dose better than first IMRT plans (*p =* 0.009). For patients whose primary GTV decreased by more than 30 cc, second IMRT plans significantly reduced the treated bowel volume [7]. In another study, the mean cervical volume reduction was 62.3% after 45 Gy EBRT [8]. In view of tumor regression, cervical cancer patients treated with IMRT received a second CT simulation and IMRT plan after 36 Gy EBRT and one to two fractions of ICBT at our institution. If we found that the tumor or cervical size had dramatically decreased before the administration of 36 Gy EBRT and one to two fractions of ICBT by CBCT or MVCT, the patient received a second CT simulation and planning in advance.

Our univariate analysis revealed that pelvic LNM was associated with worse OS and DFS. However, this finding was not significant in multivariate analysis. In contrast, pelvic LNM has correlated with worse survival in previous studies [20, 25]. A possible explanation for this is that a sufficient dose (59–61 Gy) was prescribed to positive lymph nodes with our IMRT-simultaneous-integrated-boost technique, and an extended field was used for patients with para-aortic LNM or a high risk of para-aortic lymph node failure. Moreover, the use of IGRT made the boost more accurate. It was reported that for poorly responsive lymph nodes, a total dose of >58 Gy might reduce the incidence of lymph node recurrence [26]. In the study of Vargo *et al.*, 61 cervical cancer patients (stage IB1–IVA) with PET-avid pelvic lymph nodes were treated with extended-field IMRT (45 Gy in 25 fractions and a concomitant boost to a median of 55 Gy for positive lymph nodes). Only three patients (4.9%) experienced regional node failure [27]. Kim *et al.* reported that after a median EQD2 of 62.6 Gy was delivered to bulky lymphadenopathies of cervical cancer patients through tomotherapy, final CR was observed in 52 of 58 lymph nodes, and the final lymph node response (CR vs. non-CR) was a significant prognostic factor for OS (*p* = 0.016) [28]. In our study, only 4/74 patients (5.4%) with positive pelvic lymph nodes experienced regional lymph node failure, similar to the percentage in the previous study [27]. Thus, patients with positive lymph nodes may benefit from image-guided IMRT.

In conclusion, IMRT combined with high-dose ICBT resulted in good survival and acceptable toxicity for stage IIB cervical cancer patients.

## MATERIALS AND METHODS

### Patients

We performed a retrospective study of cervical cancer patients treated with IMRT-based CCRT at our institute between May 2005 and December 2013. The inclusion criteria were as follows: biopsy-diagnosed cervical cancer; FIGO stage IIB disease; no previous surgery or radiotherapy for cervical cancer; EBRT delivered with IMRT; and concurrent chemotherapy.

### Radiotherapy

Patients were immobilized with thermoplastic in the supine position. A CT (16-slice Philips Brilliance Big Bore CT) simulation was performed with intravenous and oral contrast agents at a slice thickness of 5 mm. Vaginal marker and rectum, bladder preparation and were performed before CT simulation. The GTV and CTV were contoured on the CT images of each patient. The GTVnd included involved regional lymph nodes (including pelvic and para-aortic lymph nodes). The criteria for positive lymph nodes were a short diameter longer than 1 cm, and proof by a functional imaging technique such as PET/CT or diffusion-weighted MRI. The CTV covered the gross tumor, cervix, uterus, parametrium, upper part of the vagina to 3 cm below the tumor invasion, and pelvic lymph nodes (including the common iliac, internal iliac, external iliac, obturator and presacral lymph nodes). Patients with para-aortic LNM, bilateral pelvic LNM or involved common iliac lymph nodes received extended-field irradiation with the CTV up to the level of T12 to L1. A 5 mm margin was added to the GTVnd to create the planning gross tumor volume (PGTVnd). The planning target volume (PTV) was defined as the CTV plus an 8–10 mm margin and an additional 5–10 mm margin to the vagina, cervix and uterus.

A dose of 50.4 Gy in 28 fractions was delivered to the PTV. For patients with positive regional lymph nodes, 59–61 Gy was prescribed to the PGTVnd with the simultaneous integrated boost technique. At least 95% of the final PTV or PGTV received 100% of the prescribed dose, and at least 100% of the CTV or GTV received 100% of the dose.

The EBRT technique included FF-IMRT, VMAT and HT. All three techniques were delivered with 6-MV photons. The FF-IMRT plan was generated with seven or nine coplanar step-and-shoot beams. VMAT plans were optimized with two full arcs. The HT planning parameters were a 2.5 cm field width, a pitch of 0.25 and a maximum modulation factor of 2.5.

CBCT was performed weekly for patients receiving FF-IMRT and VMAT. Patients treated with HT received daily MVCT. CBCT or MVCT was used to determine necessary corrections to the patient’s position. Considering tumor regression, we conducted the CT simulation and IMRT planning again after 36 Gy in 20 fractions of EBRT and one to two fractions of ICBT. If the weekly CBCT or daily MVCT detected apparent motion or regression of the tumor or cervix before this time, a second round of CT simulation and treatment planning was conducted for this patient in advance.

High-dose-rate ICBT was delivered with an Ir192 resource. ICBT usually began after three weeks of EBRT. A dose of 30–36 Gy in five to seven fractions was prescribed to point A, according to the International Commission of Radiation Units 38. Patients received a conventional simulation with orthogonal films and brachytherapy planning after every insertion. A CT scan was performed after the first insertion to check the position of the applicator, identify uterine perforation and assist with 2D brachytherapy planning. Subsequently, the applicator was positioned based on the direction, curvature and depth of the tandem of the first insertion. If the doctors suspected that the uterus had been perforated, a CT scan was performed.

### Chemotherapy

All patients received concurrent chemotherapy. The first-line regimen was cisplatin 30–40 mg/m^2^ weekly. For patients with renal failure, paclitaxel 60–80 mg/m^2^ weekly was recommended.

### Follow-up and evaluation of toxicity

Gynecological examinations and pelvic MRI/CT were performed one month after treatment. Subsequently, patients had follow-up examinations approximately every three months for the next two years, every six months for three to five years after treatment, and then once per year. Treatment efficacy was assessed with the Response Evaluation Criteria in Solid Tumors. Toxicity was evaluated with the Common Terminology Criteria for Adverse Events version 3.0.

### Statistical analysis

OS, DFS and LC were estimated by the Kaplan-Meier method. Statistical significance was examined with a log-rank test. Cox’s proportional hazard model was used for multivariate analysis. Differences were considered statistically significant at *p <* 0.05. Statistical analysis was performed with SPSS v.19.0.

## Abbreviations

CBCT: cone-beam computed tomography
CCRT: concurrent chemoradiotherapy
CI: confidence interval
CRT: conformal radiation therapy
CTV: clinical target volume
DFS: disease-free survival
EBRT: external beam radiation therapy
EQD2: equivalent dose in 2-Gy fractions
FF-IMRT: fixed-field intensity-modulated radiation therapy
FIGO: International Federation of Gynecology and Obstetrics
GTV: gross tumor volume
HR: hazard ratio
HT: helical tomotherapy
ICBT: intracavitary brachytherapy
IGBT: image-guided brachytherapy
IGRT: image-guided radiation therapy
IMRT: intensity-modulated radiation therapy
LC: local control
LNM: lymph node metastases
MRI: magnetic resonance imaging
OARs: organs at risk
OS: overall survival
PET/CT: positron emission tomography/computed tomography
PGTV: planning gross tumor volume
PTV: planning target volume
SCC Ag: squamous cell carcinoma antigen
VMAT: volumetric modulated arc therapy
